# Morphology of Phagophore Precursors by Correlative Light-Electron Microscopy

**DOI:** 10.3390/cells11193080

**Published:** 2022-09-30

**Authors:** Sigurdur Runar Gudmundsson, Katri A. Kallio, Helena Vihinen, Eija Jokitalo, Nicholas Ktistakis, Eeva-Liisa Eskelinen

**Affiliations:** 1 Molecular and Integrative Biosciences, University of Helsinki, 00790 Helsinki, Finland; 2 Biomedical Center, School of Health Sciences, University of Iceland, 101 Reykjavik, Iceland; 3 Institute of Biotechnology, University of Helsinki, 00790 Helsinki, Finland; 4 Babraham Institute, Cambridge CB22 3AT, UK; 5 Institute of Biomedicine, University of Turku, 20520 Turku, Finland

**Keywords:** autophagy, phagophore, isolation membrane, omegasome, ATG13, DFCP1, ATG2, correlative light-electron microscopy

## Abstract

Autophagosome biogenesis occurs in the transient subdomains of the endoplasmic reticulum that are called omegasomes, which, in fluorescence microscopy, appear as small puncta, which then grow in diameter and finally shrink and disappear once the autophagosome is complete. Autophagosomes are formed by phagophores, which are membrane cisterns that elongate and close to form the double membrane that limits autophagosomes. Earlier electron-microscopy studies showed that, during elongation, phagophores are lined by the endoplasmic reticulum on both sides. However, the morphology of the very early phagophore precursors has not been studied at the electron-microscopy level. We used live-cell imaging of cells expressing markers of phagophore biogenesis combined with correlative light-electron microscopy, as well as electron tomography of ATG2A/B-double-deficient cells, to reveal the high-resolution morphology of phagophore precursors in three dimensions. We showed that phagophores are closed or nearly closed into autophagosomes already at the stage when the omegasome diameter is still large. We further observed that phagophore precursors emerge next to the endoplasmic reticulum as bud-like highly curved membrane cisterns with a small opening to the cytosol. The phagophore precursors then open to form more flat cisterns that elongate and curve to form the classically described crescent-shaped phagophores.

## 1. Introduction

Macroautophagy, hereafter called autophagy, is a pathway that sequesters and transports cytoplasmic material and intracellular pathogens to lysosomes for degradation and recycling [1]. Autophagy can rapidly respond to a multitude of different cellular stress stimuli, such as starvation. Amino acid starvation causes the inactivation of mammalian target of rapamycin (mTOR), which leads to the partial dephosphorylation of autophagy-related 13 (ATG13). This initiates the activation of a protein kinase complex (the ULK1 complex), consisting of Unc-51-like autophagy activating kinase 1 (ULK1), ATG13, FAK family kinase-interacting protein of 200 kDa (FIP200, also called RB1CC1) and autophagy-related 101 (ATG101) [2,3]. The active ULK1 complex is recruited to the endoplasmic reticulum (ER), followed by the phosphoinositide-3-kinase (PI3K) complex consisting of PI3K class 3 (PI3KC3, also called VPS34), phosphoinositide-3-kinase regulatory subunit 4 (PIK3R4/VPS15), ATG14 and Beclin 1. These complexes are followed by other elements of the machinery required for autophagosome nucleation, elongation and closure [4].

Autophagosome biogenesis includes the translocation of the double FYVE domain-containing protein 1 (DFCP1) to the ER. DFCP1 forms punctate ER subdomains that expand into circular ER subdomains called omegasomes [5]. Phagophores (also called isolation membranes) nucleate and elongate inside omegasomes. When the phagophore reaches maturity, it fuses to form a double-membraned autophagosome, and the DFCP1-positive omegasome shrinks and disappears. Autophagosomes subsequently fuse with endosomes to form amphisomes [6], where acidification starts. Amphisomes eventually fuse with lysosomes to form autolysosomes [7], where the bulk of the cargo degradation occurs, and the metabolites are then recycled through the autolysosomal membrane back to the cytoplasm.

Phagophores have also been demonstrated to nucleate from recycling endosomes and subsequently make membrane contact sites (MCSs) with other organelles, mainly the ER, in order to elongate [8,9,10]. Multiple other organelles have also been reported as membrane sources for the phagophores, such as mitochondria [11] and the Golgi complex [12,13,14]. There is also evidence for de novo lipid synthesis during phagophore biogenesis [15].

Recent results show that ATG2, located at the ER–phagophore MCS, facilitates lipid transfer from the ER to the phagophore via a hydrophobic channel in the protein [16,17,18,19]. Recent papers have resolved the structure of the autophagy protein ATG9 in yeast and mammals. The structure shows that ATG9 contains a hydrophobic groove, similar to ATG2B. ATG9 was shown to function as a scramblase by transferring lipids from the outer to the inner membrane leaflet in the growing phagophore membrane [20,21]. ATG2 and ATG9 are both required for phagophore expansion: ATG2 transfers lipids from the ER to the outer membrane leaflet, and ATG9 then distributes the lipids to the inner membrane leaflet during phagophore expansion. 

We and others demonstrated that autophagosome biogenesis occurs in a close relationship with the ER, with rough ER cisterns typically lining the elongating phagophores on both sides [22,23]. However, despite the recent great advances in clarifying the molecular events during phagophore initiation [24], the morphological events during the early phases of phagophore biogenesis are still not fully understood. To elucidate the first steps of phagophore biogenesis at high resolution in three dimensions (3D), we used live-cell imaging and correlative light-electron microscopy (CLEM) to visualize the structure of the phagophore precursors at different stages of formation. This approach enabled us to identify the nascent phagophores in live cells expressing fluorescently labeled DFCP1 or ATG13 proteins involved in the initial steps of phagophore biogenesis [4,5]. CLEM and 3D electron microscopy were then used to image the membrane morphology of the forming phagophores. We also utilized 3D electron microscopy of cells deficient in ATG2, which have been shown to accumulate phagophore precursors [25]. Our results show the high-resolution morphology of phagophore precursors and demonstrate their intimate relationship with the ER, as well as with vesicles of differing morphologies.

## 2. Materials and Methods

### 2.1. Live-Cell Imaging for CLEM

HEK293 cells stably expressing GFP-DFCP1 or GFP-ATG13 (Karanasios et al., 2013) were grown on gridded MatTek glass-bottom dishes to subconfluency. The cells were placed in starvation medium (140 mM NaCl, 1 mM CaCl_2_, 1 mM MgCl_2_, 5 mM glucose, 1% bovine serum albumin and 20 mM Hepes (pH 7.4)) before being placed on the light microscope stage kept at +37 °C with 5% CO_2_. Cells with DFCP1 or ATG13 puncta were time-lapse imaged after 15–30 min starvation. Target cells were imaged every 15 s until a 15 min image sequence was captured, using a 100× 1.4 NA objective on a 3I Marianas spinning-disc confocal microscope or a GE DeltaVision ultrawide-field fluorescence microscope. Subsequently, 50–75% of the starvation medium was replaced with 4% paraformaldehyde in 0.2 M Hepes buffer (pH 7.4). The fixative mixture was then replaced with fresh 4% paraformaldehyde in 0.2 M Hepes buffer. After a 10 min incubation, the fixative was replaced with 0.2 M Hepes buffer to allow the capture of a Z stack using the 100× 1.4 NA objective. The MatTek-dish grid coordinates and cells were then imaged with a 20× phase-contrast objective.

### 2.2. CLEM with Serial Block-Face-Imaging Scanning Electron Microscopy (SBEM)

After live-cell imaging, the cells were fixed again in 2.5% glutaraldehyde (Sigma, G5882) in 0.1 M sodium cacodylate buffer (pH 7.4), supplemented with 2 mM CaCl_2_ and postfixed using double osmication prior to uranyl acetate en bloc staining and lead aspartate treatment, as described previously [26]. The method was modified from [27]. Finally, the cells were dehydrated and embedded in resin (Fluka, Durcupan ACM) between two objective glasses. After polymerization, the target area was cut out using a razor blade, according to the coordinates obtained from the phase-contrast microscopy. The block faces were imaged with 2.5 kV acceleration voltage, 0.3 Torr pressure and a spot size of 3 using a backscattered-electron detector (Gatan Inc., 25650-30, Pleasanton, CA, USA) in a FEG-SEM Quanta 250 microscope (FEI Company, Hillsboro, OR, USA). The microscope chamber was equipped with an ultramicrotome (3View, Gatan Inc., Pleasanton, CA, USA), which allowed for the sectioning (30 nm sections) of the block face. Images of the block face were captured using Gatan Digital Micrograph software, and the captured images were aligned, cropped and normalized using Microscope Image Browser (MIB) [28]. Segmentation was performed with MIB, and the 3D models were visualized using 3D Slicer [29]. The image stacks were filtered by a deep-neural-network denoising filter in 3D mode using MIB.

### 2.3. CLEM with Electron Tomography

After live-cell imaging, the samples were fixed again in 2% glutaraldehyde in 0.2 M Hepes buffer (pH 7.4) for 30 min. The samples were then postfixed in 1% OsO_4_, 0.1 M sodium cacodylate (pH 7.4) containing15 mg/mL potassium ferrocyanide at room temperature for 1 h, washed with 0.1 M sodium cacodylate buffer and water and incubated with 1% uranyl acetate at 4 °C for 1 h. After washing, the samples were dehydrated in ethanol series (50%, 70%, 96% and 100%), infiltrated in resin (TAAB 812, T030), flat-embedded, and polymerized at +60 °C overnight. A pyramid was trimmed using a razor blade according to the coordinates obtained from the light microscopy. The samples were cut using a diamond knife to semithick serial 230 nm sections for electron tomography. The sections were picked up on single-slot grids. For electron-tomogram alignment, 10 nm gold particles were applied on both sides of the 230 nm sections. Dual-axis tilt series were acquired using SerialEM software [30] on a Tecnai FEG20 transmission electron microscope (FEI, the Netherlands) operating at 200 kV and 11500X primary magnification, over a tilt range of ±62 degrees. Tilt series were aligned and reconstructed into tomograms using IMOD [31], and segmented using MIB [28]. The 3D models were visualized using 3D Slicer [29] or Amira software (Visage Imaging Inc., San Diego, CA, USA, version 5.3.2).

Image processing for CLEM was performed by deconvoluting the fluorescence Z-stack images using Microvolution or Deltavision deconvolution algorithms. The images were then correlated using the TrakEM2 [32] module of Fiji [33] by transforming the fluorescence image and superimposing it on top of a low-magnification EM image of the cell of interest.

### 2.4. Electron Tomography of ATG2A and ATG2B Double-Knockout Cells

ATG2A and ATG2B double-knockout mouse fibroblasts [34] were a kind gift of Noboru Mizushima, The University of Tokyo, Japan. The cells were fixed in 2% glutaraldehyde in 0.2 M Hepes (pH 7.4) for 30 min, and they were processed for resin embedding and semithick sectioning using the protocol described above for CLEM tomography. Acquisition of tilt series and reconstruction of tomograms were performed as described above. Tomograms were segmented using the manual drawing tool in MIB, except for ferritin, which was automatically segmented using local threshold levels. The 3D models were visualized using Amira software. 

### 2.5. Fluorescence Microscopy

HEK293 cells stably overexpressing GFP-ATG13 were cultured on glass coverslips for 48 h and then incubated in starvation medium (140 mM NaCl, 1 mM CaCl_2_, 1 mM MgCl_2_, 5 mM glucose, 1% bovine serum albumin and 20 mM Hepes (pH 7.4)) for 60 min. The cells were fixed in 4% paraformaldehyde in 0.2 M Hepes buffer (pH 7.4) for 10 min, permeabilized in 0.1% Triton X-100 in phosphate-buffered saline (PBS) (pH 7.4) for 10 min and blocked with 1% bovine serum albumin at room temperature for 3 h. The cells were labeled with mouse monoclonal anti-human transferrin receptor (TfR) (Invitrogen 136,800) at 1:200 dilution, or anti-ATG2B (Thermo Fisher 25155-1-AP) at 1:200 dilution, in 0.1% bovine serum albumin in PBS at +4 °C overnight. After washing in PBS, the cells were labeled with Alexa Fluor 647-conjugated goat-anti-mouse IgG secondary antibody (Thermo Fisher A32728). The coverslips were embedded onto microscopy slides with ProLong™ Glass Antifade Mountant with NucBlue™ Stain (Thermo Fisher P36981) antifade mounting medium, and they were imaged using a GE Deltavision ultrawide-field fluorescence microscope.

Images were deconvoluted using the Deltavision deconvolution algorithm, and then segmented using Imaris spot detection. GFP-ATG13 puncta with epicenters located within 120 nm from the epicenters of puncta positive for TfR or ATG2B were quantified and expressed as a proportion of the total ATG13 puncta located in proximity to TfR- or ATG2B-positive puncta.

## 3. Results

### 3.1. Phagophore Assembly in Relation to the DFCP1-Positive Omegasome

Earlier live-cell-imaging experiments suggested that, at the time point when the omegasome diameter is at its largest (~1 µm), the nascent phagophore inside the omegasome is still open and expanding, while the closure of the autophagosome takes place after the omegasome diameter has decreased [4,5]. In order to clarify the phagophore morphology in relation to the omegasome, we used CLEM to visualize the phagophores in HEK293 cells expressing the omegasome marker protein DFCP1 tagged with GFP. First, we used live-cell imaging, which was followed by serial block-face imaging scanning electron microscopy (SBEM). The time-lapse videos were used to locate the omegasomes at their expanded stage, and those locations were then traced in the SBEM image stacks. We observed that the phagophores located in proximity to the large omegasomes were already expanded, typically elongated, closed or nearly closed autophagosomes (Supplementary Figures S1 and S2). Furthermore, mitochondria were observed in close proximity to the phagophores/autophagosomes.

**Figure 1:**
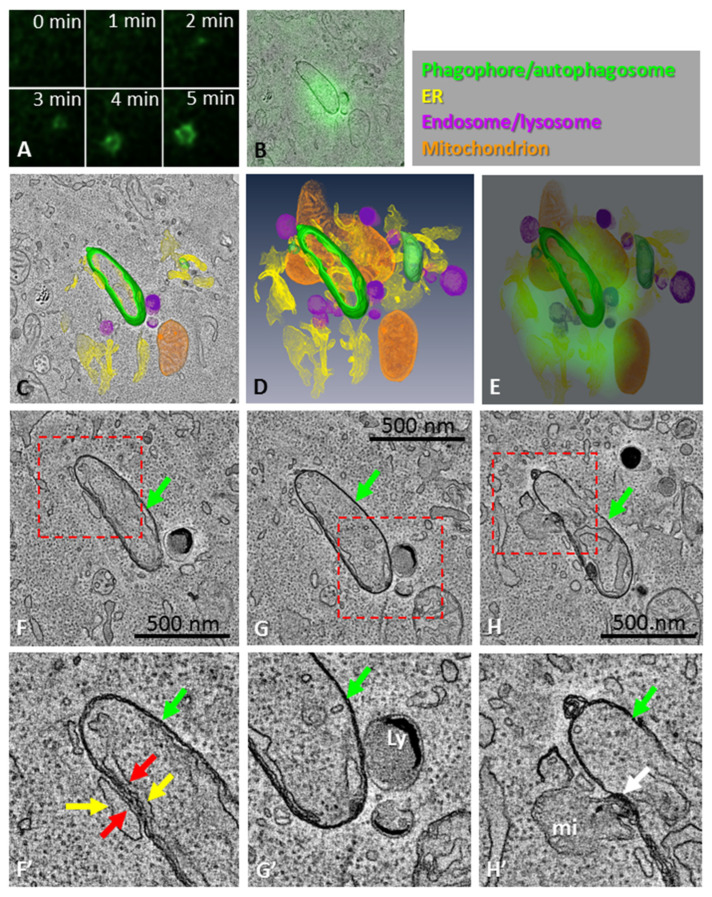
CLEM of an omegasome and a nascent phagophore/autophagosome. (**A**) HEK293 cells expressing GFP-tagged DFCP1 were time-lapse imaged to trace omegasomes. After fixation, the same cell and omegasome were imaged with electron tomography. (**B**) One slice of the tomogram overlaid with the DFCP1 fluorescence. (**C**) The phagophore, ER, lysosomes and mitochondria were traced to create a 3D model, using the color code shown in the top right corner of the figure. Panel (**C**) shows one tomography slice overlayed with part of the 3D model. (**D**) The 3D model shows the relationships of the phagophore, ER, mitochondria and lysosomes with each other. (**E**) The DFCP1 fluorescence from live-cell imaging overlaid with the 3D model. Note that the DFCP1 localization overlaps the ER in the 3D model. (**F**–**H**) Slices through the tomogram. Panels (**F’**–**H’**) show the boxed areas at higher magnification. (**F**,**F’**) The phagophore/autophagosome (green arrows) has MCSs (red arrows in (**F’**)) with the ER (yellow arrows) inside and outside of the phagophore. (**G**,**G’**) The phagophore/autophagosome (green arrows) has MCSs with a lysosome (Ly). (**H**,**H’**) The phagophore/autophagosome (green arrows) also has an MCS (white arrow in (**H’**)) with a mitochondrion (mi). In panels (**D**,**E**), ER, mitochondria and lysosomes are shown as volume rendered according to their grey-level values. See also Supplementary Figure S1.

In order to acquire higher-resolution images, we used live-cell imaging followed by electron tomography. As with SBEM, we observed that the phagophores or autophagosomes associated with large omegasomes were elongated in shape (Figure 1A–H, Supplementary Figure S1). The elongated shape has been described to correspond to phagophores just before they close to become autophagosomes [35]. When the 3D-modelled ER structure in the tomogram was correlated with the omegasome DFCP1 fluorescence, their sizes and shapes matched (Figure 1B,E), indicating that the DFCP1-positive omegasome consisted of ER sheets and tubules surrounding the phagophore assembly site, as suggested before using live-cell imaging [5]. The phagophore/autophagosome next to the omegasome in the tomogram showed MCSs with the ER on both the inside and outside of the phagophore/autophagosome (Figure 1F,F´), as well as with a mitochondrion (Figure 1H,H´). Notably, the phagophore/autophagosome also had an MCS with a lysosome (Figure 1G,G´), suggesting the phagophore was close to forming a sealed autophagosome, or already closed into an autophagosome. These findings suggested that the phagophores are already maximally elongated, closed or nearly closed, at the stage when the omegasome diameter is still large. This implies that the phagophore closes before the omegasome collapses.

### 3.2. Phagophore Precursors in ATG2A/B-Double-Deficient Mouse Embryonic Fibroblasts

**Figure 2:**
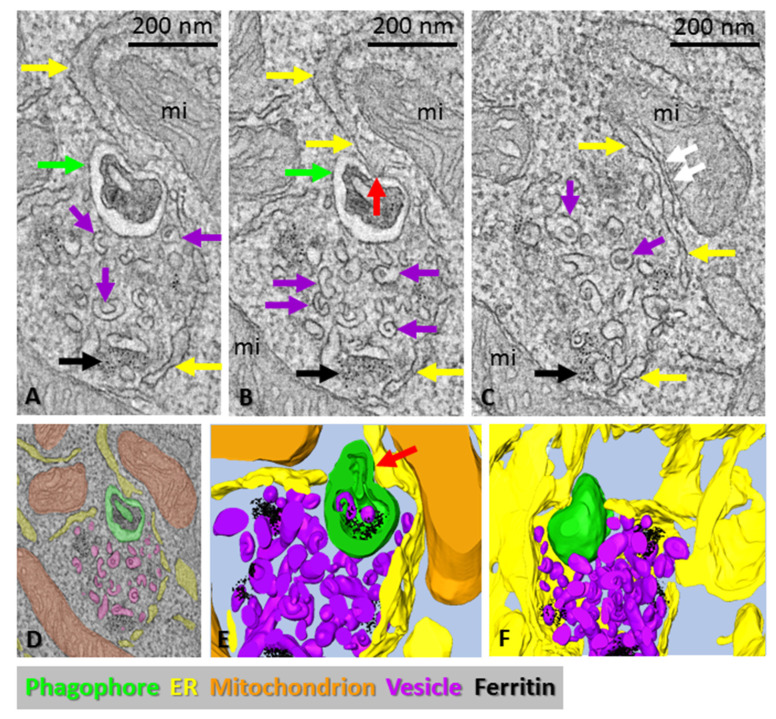
Electron tomography of a mouse embryonic fibroblast deficient in ATG2A and ATG2B. (**A**–**C**) Tomography slices of a ribosome-free area containing a double-membrane vesicle (a putative phagophore precursor (green arrows)), cup-shaped vesicles (purple arrows), ferritin particles (black arrows) and surrounded by ER (yellow arrows) and mitochondria (mi). The red arrow in panel (**B**) indicates an MCS between the phagophore precursor and the ER, and the white arrows in panel (**C**) point to an MCS between the ER and a mitochondrion. (**D**) Organelles in the tomogram were traced with different colors, as indicated by the overlay, as well as the bottom panel of the figure. (**E**,**F**) Details of the 3D model. Ferritin particles are shown in black. Note the MCS between the phagophore precursor and ER (red arrow), also visible in panel (**B**). The phagophore precursor contains ferritin and two vesicles.

ATG2 has been shown to localize at ER–phagophore MCSs and transfer lipids from the ER to the expanding phagophore [16,17,18,19]. Kishi-Itakura et al. showed that ATG2-deficient cells accumulate specific ribosome-free areas lined by the ER that are rich in 50–60 nm vesicles and that contain clusters of ferritin particles and 200–300 nm double-membraned structures positive for the autophagosome marker LC3 [25]. They also showed that at least part of the 50–60 nm vesicles in the ribosome-free areas were positive for ATG9 [25]. Because ATG2 was recently shown to function as a lipid-transfer protein during phagophore biogenesis [16,17,18,19], we hypothesized that the 200–300 nm double-membrane vesicles in ATG2-deficient cells are phagophore precursors. We used ATG2A/ATG2B-double-deficient mouse embryonic fibroblasts for electron tomography in order to define the 3D morphology of the ribosome-free areas (Figure 2). The tomograms revealed spherical double-membrane structures with diameters of approximately 200 nm (Figure 2A,B,D–F). We also observed MCSs between the double-membrane structures and the ER (Figure 2B,E). The ribosome-free areas were rich in cup-shaped 50–60 nm vesicles (Figure 2A–F). Ferritin particles were also visible in the ribosome-free areas (Figure 2), as also reported by Kishi-Itakura et al. [25].

To summarize, the ATG2A/B-deficient cells are able to form phagophore precursors/small autophagosomes of approximately 200 nm in diameter in the regions rich in cup-shaped 50–60 nm vesicles. 

### 3.3. ATG13-Positive Phagophore Precursor Localizes in Proximity to ATG2 but Not TfR 

ATG13 is a subunit of the ULK1 complex that initiates phagophore biogenesis by translocating to the ER [4,36]. In order to visualize the earliest steps of phagophore biogenesis with correlative light-electron microscopy (CLEM), we used HEK293 cells expressing GFP-ATG13, which were used earlier to demonstrate that GFP-ATG13 puncta mark sites of phagophore biogenesis [4]. In addition to the ER, recycling endosomes have also been implicated in the nucleation or biogenesis of phagophores [8,9,10,37,38]. As stated above, ATG2 localizes at the ER–phagophore MCSs, where it transfers lipids from the ER to the expanding phagophore [16,17,18,19]. To clarify whether the ATG13-positive phagophore precursors in the HEK293 cells interact with the ER and/or recycling endosomes, we performed immunofluorescence staining of the GFP-ATG13 cells for ATG2 (marker for ER–phagophore contacts) or transferrin receptor (TfR) (marker for recycling endosomes). We automatically segmented the fluorescent puncta of GFP-ATG13, ATG2 and TfR, and quantified the ATG2 and TfR puncta with epicenters within 120 nm from the epicenters of GFP-ATG13 puncta (Supplementary Figure S3). The analyses were performed for cells kept under full-medium conditions, amino acid starvation and amino acid starvation in the presence of SAR405, a PIK3C3/VPS34 inhibitor, which is known to prevent phagophore biogenesis. Under amino acid starvation, the percentage of ATG13 puncta colocalizing or in proximity to ATG2 slightly increased from approximately 34% in the full medium to 50% in the amino acid-free medium, although the difference was not statistically significant. However, the inhibition of PI3K activity with SAR405 in the amino acid-free medium decreased the colocalization to 15% (Supplementary Figure S3). Although amino acid starvation also slightly, but not statistically significantly, increased the colocalization/proximity localization of GFP-ATG13 and TfR, the colocalization was only 17% under amino acid-free conditions. SAR405, nevertheless, decreased the colocalization to 9%. These results suggest that the GFP-ATG13-positive phagophore precursors interact with the ER, but they are less likely to interact with recycling endosomes. This does not rule out that phagophores can also interact with recycling endosomes; however, the system we used was less likely to track this population.

### 3.4. Morphology of Phagophore Precursors and Phagophores in Amino Acid-Starved HEK293 Cells

**Figure 3:**
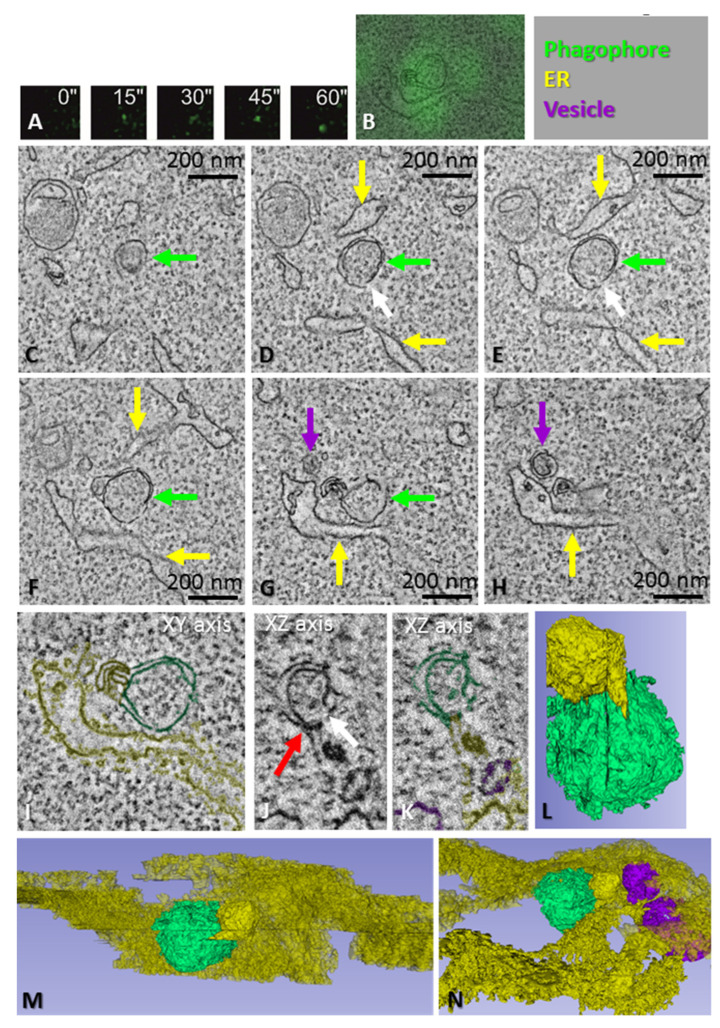
CLEM of a phagophore precursor. (**A**) HEK293 cells expressing GFP-tagged ATG13 were time-lapse imaged to trace ATG13 puncta. The cells were fixed 60 s after the appearance of the ATG13 punctum. After fixation, the same cell and punctum were imaged with electron tomography. (**B**) One slice of the tomogram overlaid with the ATG13 fluorescence. (**C**–**H**) Tomography slices showing the phagophore precursor (green arrows), ER (yellow arrows) and cup-shaped vesicles (purple arrows). The white arrows in panels (**D**,**E**,**J**) indicate a small opening in the phagophore precursor. Note the phagophore precursor contains ribosomes, similar to the cytoplasm around it. (**I**) Organelles in the tomogram were traced with different colors, as indicated by the overlay, as well as the top right panel of the figure. (**J**,**K**) Tomography slice showing the phagophore precursor and ER from a different angle compared with panels (**B**–**I**). Note the MCS between the phagophore precursor and ER (red arrow in panel (**J**)), and the small opening in the phagophore precursor (white arrow in panel (**J**)). (**L**–**N**) Views of the 3D model from different angles, demonstrating the close apposition of the phagophore precursor and ER, as well as the vesicles. See also Supplementary Figure S1.

**Figure 4:**
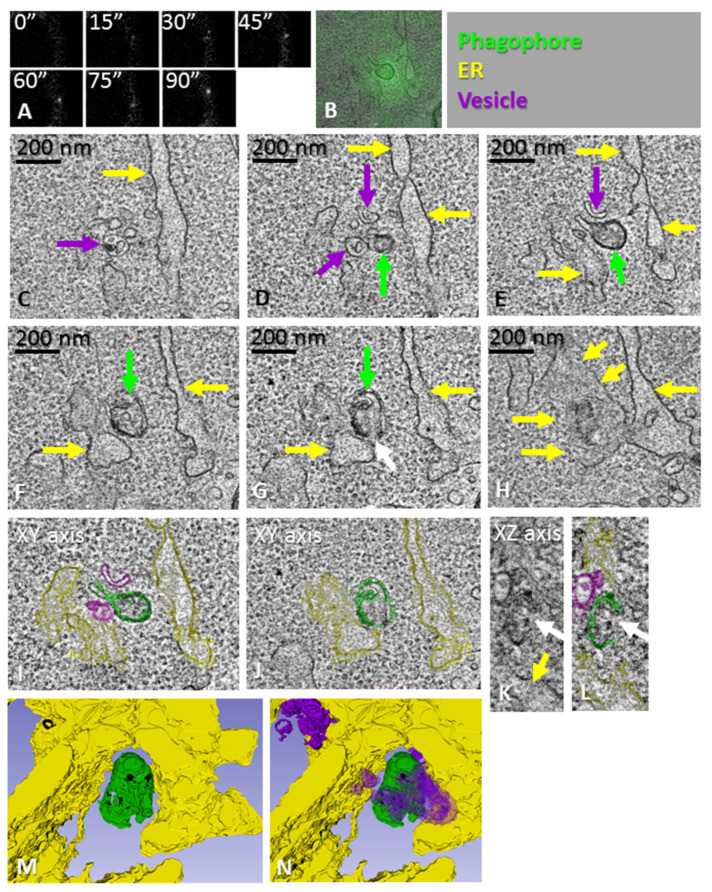
CLEM of a phagophore precursor. (**A**) HEK293 cells expressing GFP-tagged ATG13 were time-lapse imaged to trace ATG13 puncta. The cells were fixed 90 s after the appearance of the ATG13 punctum. After fixation, the same cell and punctum were imaged with electron tomography. (**B**) One slice of the tomogram overlaid with the ATG13 fluorescence. (**C**–**H**) Tomography slices showing the phagophore precursor (green arrows), ER (yellow arrows) and cup-shaped vesicles (purple arrows). The white arrow in panel (**G**) indicates a small opening in the phagophore precursor. (**I**,**J**) Organelles in the tomogram were traced with different colors, as indicated by the overlay, as well as the top right panel of the figure. (**K**,**L**) Tomography slice showing the phagophore precursor, ER and one vesicle from a different angle compared with panels (**C**–**J**). (**M,N**) Views of the 3D model from different angles, demonstrating the close apposition of the phagophore precursor and ER, as well as the vesicles. See also Supplementary Figure S1.

In order to visualize the earliest steps of phagophore biogenesis with correlative light-electron microscopy (CLEM), HEK293 cells expressing GFP-ATG13 were first imaged live under a fluorescence microscope to trace newly formed ATG13 puncta. CLEM was then used to trace these puncta in 3D electron microscopy. When the ATG13 puncta were fixed within the first 1–3 min of their lifetimes and imaged using electron microscopy, they corresponded to spherical structures with a diameter of approximately 200 nm, expanding from an ER sheet or tubule (Figure 3 and Figure 4). Tomograms showed that, instead of being flat cisterns that elongate and bend, the phagophore precursors were highly curved structures with a small opening to the cytoplasm (Figure 3D,E; Figure 4G,L). Cup-shaped vesicles were observed next to the phagophore precursors (Figure 3G,H; Figure 4C–E). The phagophore precursors had a close relationship with the ER, and MCSs with the ER were observed (Figure 3E,G,H,J–N; Figure 4G,J–L). In summary, both the double-membrane structures in the ATG2A/B-deficient cells and the ATG13-positive phagophore precursors in the starved HEK293 cells were surrounded by the ER, had MCSs with the ER and were located next to cup-shaped vesicles.

**Figure 5:**
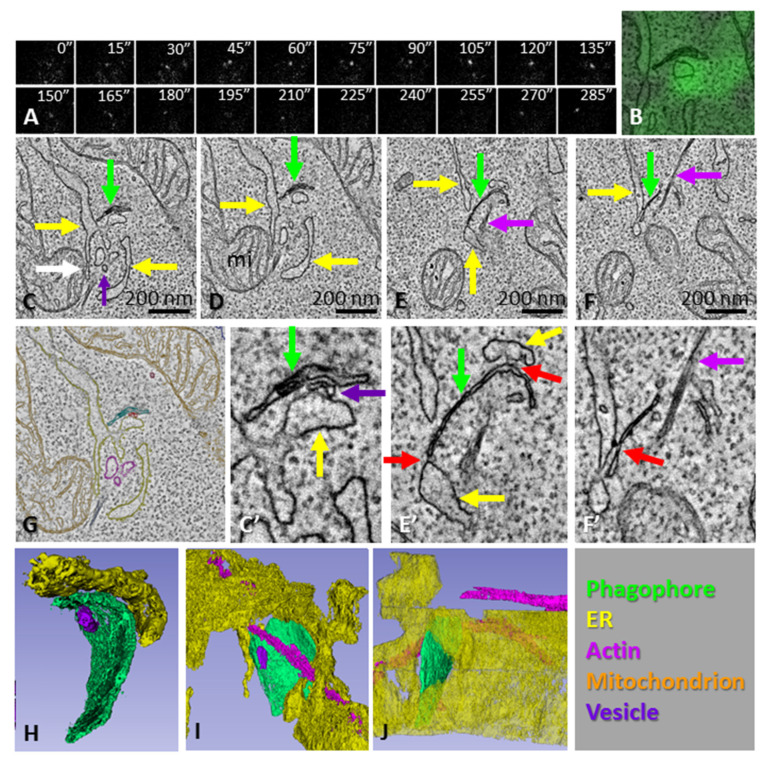
CLEM of a phagophore. (**A**) HEK293 cells expressing GFP-tagged ATG13 were time-lapse imaged to trace ATG13 puncta. The cells were fixed 4 min 45 s after the appearance of the ATG13 punctum. After fixation, the same cell and punctum were imaged with electron tomography. (**B**) One slice of the tomogram overlaid with the ATG13 fluorescence. (**C**–**F**) Tomography slices showing the phagophore (green arrows), ER (yellow arrows), vesicles (purple arrows) and a bundle of putative actin filaments (pink arrows). The white arrow in panel (**C**) indicates an MCS between a mitochondrion (mi) and ER close to the phagophore. (**G**) Organelles in the tomogram were traced with different colors, as indicated by the overlay, as well as the bottom right panel of the figure. Panels (**C’**,**E’**,**F’**) show enlarged details from panels (**C**,**E**,**F**), respectively. The red arrows in panels (**E’**,**F’**) indicate MCSs between the phagophore and ER. (**H**–**J**) Views of the 3D model from different angles, demonstrating the close apposition of the phagophore, ER and vesicles, as well as the location of the actin filament bundle. See also Supplementary Figure S1.

Figure 5 shows the morphology of an ATG13 punctum that was fixed after 4 min 45 s after its formation, suggesting that the phagophore was more mature than the examples in Figure 3 and Figure 4. Tomography of this punctum revealed an open slightly bent phagophore (Figure 5E,E´,H) that showed MCSs with the ER at the edge of the cistern and with the convex surface of the curved cistern (Figure 5E,E´,F,F´). A bundle of filaments, likely actin filaments, were located next to the phagophore (Figure 5F,F´,I,J).

These findings suggest that phagophores emerge next to the ER as spherical highly curved structures of approximately 200 nm in diameter, which subsequently open into flat or less bent cisterns that elongate until they bend and close into autophagosomes. Initially, the phagophore precursor forms a single MCS with the ER, but as the phagophore expands, it can form additional MCSs with the ER and other organelles, which might correspond to multiple lipid-transfer sites to the elongating phagophore.

## 4. Discussion

In this study, we visualized phagophore biogenesis in relation to the ER subdomain, which is called the omegasome. Our data suggest that when the DFCP1-positive omegasome has its largest diameter, the phagophore is already fully expanded and is close to fusing or is already fused into an autophagosome. The omegasome explains how the ER is involved in the formation of the phagophore, and how lipids can be transported from the ER to the phagophore. The omegasome connects to the forming phagophore via thin tubules (Figure 1, Supplementary Figure S4 and [39]), and this may be the means by which ATG2 transfers lipids to the expanding phagophore. However, the state of the phagophore biogenesis during its association with the omegasome has not been determined before. Here, we show that the phagophore can be fully formed and expanded while still associated with the omegasome.

We also visualized the biogenesis of GFP-ATG13-positive phagophores at the early stages of nucleation and expansion. We observed that phagophore precursors emerged next to the ER as bud-like highly curved double-membrane structures with a small opening to the cytosol. Double-membrane structures of a similar size and morphology were also observed in cells deficient in ATG2A and ATG2B. The initial ATG13-positive phagophore precursors then opened up to form more flat cisterns, similar to the classically described phagophores/isolation membranes, which elongated and curved to eventually close up to form autophagosomes. 

Recent reports have shown that ATG2 facilitates the lipid transfer from the ER to the phagophore [16,17,18,19]. In agreement with these findings, we observed that, under autophagy-stimulating conditions, ATG13-positive and ATG2-positive puncta were proximal or colocalized. Furthermore, ATG2A/B-deficient cells were still able to form bud-like highly curved double-membraned LC3-positive phagophore precursors/small autophagosomes, while fully extended phagophores and autophagosomes were missing [25]. In these cells, phagophore elongation is likely halted due to failure in the lipid transport from the ER. We used ATG2A and ATG2B knockout fibroblasts to analyze the 3D morphology of the double-membrane structures. Their sizes and morphologies were similar to the ATG13-positive phagophore precursors we observed by CLEM in starved HEK293 cells.

Phagophores corresponding to very recently formed (1–2 min) GFP-ATG13 puncta, as well as the phagophore precursors in the ATG2A/B-deficient cells, had a single MCS with the ER, while phagophores corresponding to more advanced GFP-ATG13 puncta were more expanded and had several MCSs with the ER, which may indicate multiple lipid-transfer sites via ATG2 or other lipid-transfer systems. Additionally, the lipid transfer from the ER to the convex surface of the curving cistern might also increase the curvature of the phagophore. 

It has been suggested that mitochondria provide membranes for forming autophagosomes [11], and autophagosomes have been reported to form at ER–mitochondria contact sites [40]. However, the role of mitochondria in phagophore/autophagosome biogenesis is still poorly understood. We observed ER–mitochondrion MCSs in the vicinity of the phagophore precursors both in mouse embryonic fibroblasts deficient in ATG2A/B and in HEK293 cells. In our experiments, only the larger, extended and elongating phagophores had MCSs with mitochondria (not shown; [14]). This supports the notion that mitochondria may have a role during phagophore expansion rather than nucleation.

A recent paper showed that phagophore biogenesis initiates by the fusion of the biosynthetic and endocytic pathways in a novel organelle called the hybrid pre-autophagosomal structure (HyPAS), which is positive for the ULK1-complex component FIP200 and the core autophagy protein ATG16L1 [41]. By electron microscopy, the HyPAS morphology corresponds to a cluster of vesicles and tubules, reminiscent of the vesicular tubular morphology of the Atg9 reservoirs located next to the ER and phagophores in yeast [42]. We regularly observed 30–60 nm vesicles, many of them cup-shaped, next to the phagophore precursors in both the HEK293 cells and ATG2A/B-deficient cells. Kishi-Itakura et al. [25] showed that, in ATG2-deficient cells, similar vesicles accumulate in ribosome-free areas together with 200 nm double-membraned LC3-positive vesicles (which we call phagophore precursors). Immuno-electron microscopy showed that at least part of these cup-shaped vesicles were positive for ATG9 [25]. Other studies have reported that ATG9 vesicles can be cup-shaped and can localize in the vicinity of the ER and phagophores [43,44]. Furthermore, ATG9 vesicles are important for autophagosome biogenesis [42,45], and recent findings show that ATG9 vesicles act as seeds for autophagosome formation [46]. The current view is that ATG2 and ATG9 are both required for phagophore expansion: ATG2 transfers lipids from the ER to the outer membrane leaflet, and ATG9 then distributes the lipids to the inner membrane leaflet during phagophore expansion (reviewed in [47]). Thus, rather than being a lipid source for phagophore expansion, ATG9 vesicles may provide the machinery for lipid transfer and distribution. In addition to vesicles positive for FIP200, ATG16L1 or ATG9, other types of vesicles have also been implicated in phagophore biogenesis, including COPII vesicles [14,48,49]. The vesicles we observed in the vicinity of the phagophore precursors may be ATG16L1, FIP200 or ATG9 vesicles, uncoated COPII vesicles, or other types of vesicles. Further studies are needed to clarify this issue.

It has been suggested that actin filaments regulate autophagosome biogenesis by assembling inside the phagophore, thus contributing to the shape of the phagophore membranes [50]. Furthermore, actin-comet-tail assembly has been suggested to assist the autophagosome biogenesis in the ER, placing the actin assembly outside the phagophore [51]. We did not observe actin filaments inside or outside of the phagophore precursors. However, we did observe a bundle of putative actin filaments in proximity to an elongating phagophore (Figure 5). However, the bundle did not seem appropriately positioned for being able to guide the shape of the forming phagophore, and the orientation of the filaments did not resemble a comet tail. It is possible that the actin assemblies contributing to phagophore biogenesis were not well contrasted in the resin-embedded EM samples we used in our experiments.

## Supplementary Materials

The following supporting information can be downloaded at: https://www.mdpi.com/article/10.3390/cells11193080/s1, Figure S1: Low-magnification overlays of GFP fluorescence images and electron microscopy images; Figure S2: CLEM of an omegasome and a nascent phagophore/autophagosome using SBEM; Figure S3: Colocalization analysis of GFP-tagged ATG13 with endogenous ATG2 and TfR; Figure S4: Model of autophagosome biogenesis in the omegasome.
