# Supplementary material for: Morphology of Phagophore Precursors by Correlative Light-Electron Microscopy
Source: Cells. 2022 Sep 30;11(19):3080. doi: 10.3390/cells11193080 (PMC9562894; doi:10.3390/cells11193080)
Supplement: Supplementary file 1 [file cells-11-03080-s001.zip › cells-1846528-supplementary.pdf]

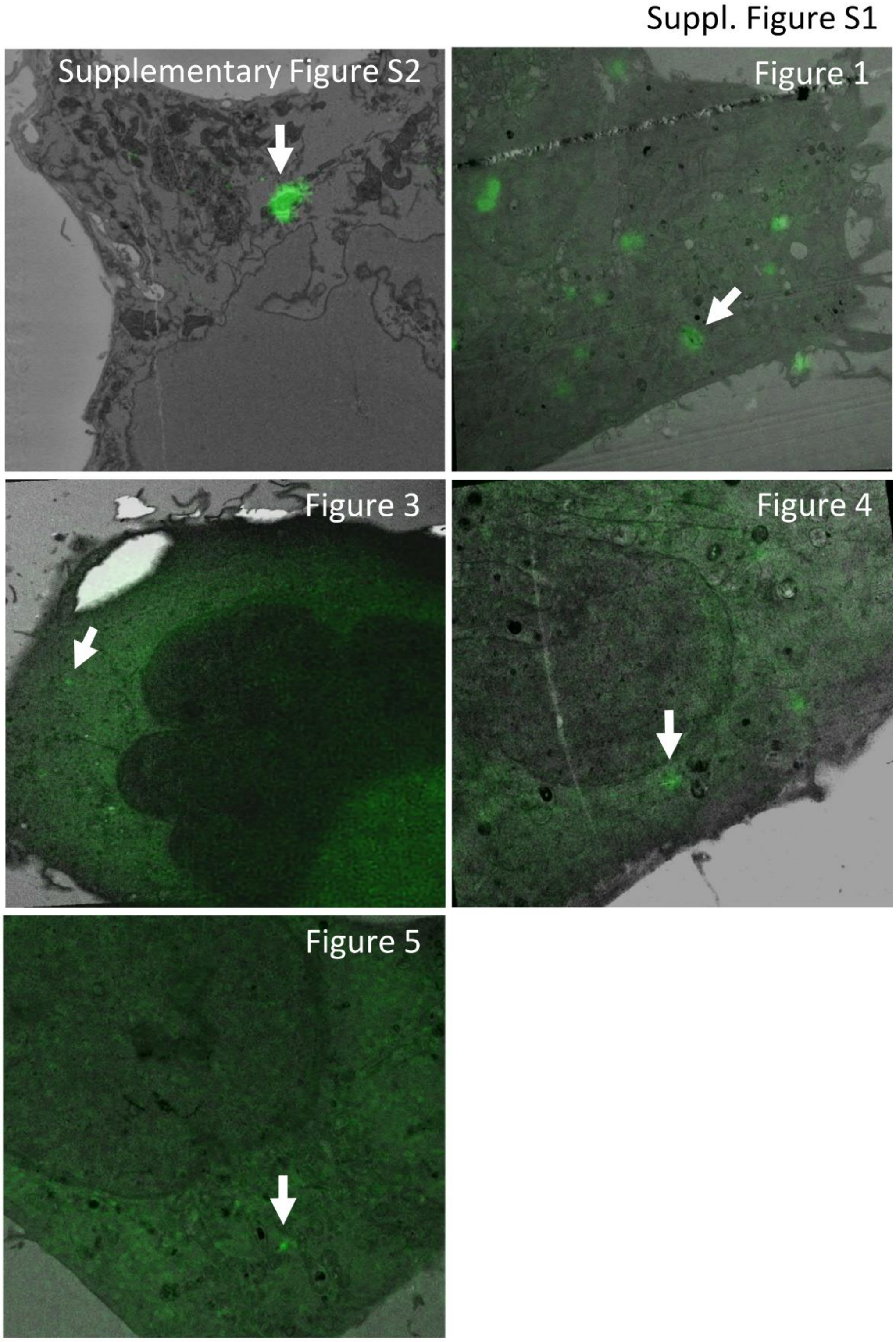

Supplementary Figure S1. Low-magnification overlays of GFP fluorescence images and electron microscopy images to complement Supplementary Figure S2, and Figures 1, 3, 4 and 5. The overlays were done using one image from the SBEM image stack (Supplemtary Figure S1), or a low-magnification image of one of the 230-nm serial sections used for tomography (Figures 1, 3, 4 and 5). The arrows indicate the structures of interest.

Suppl. Figure S2

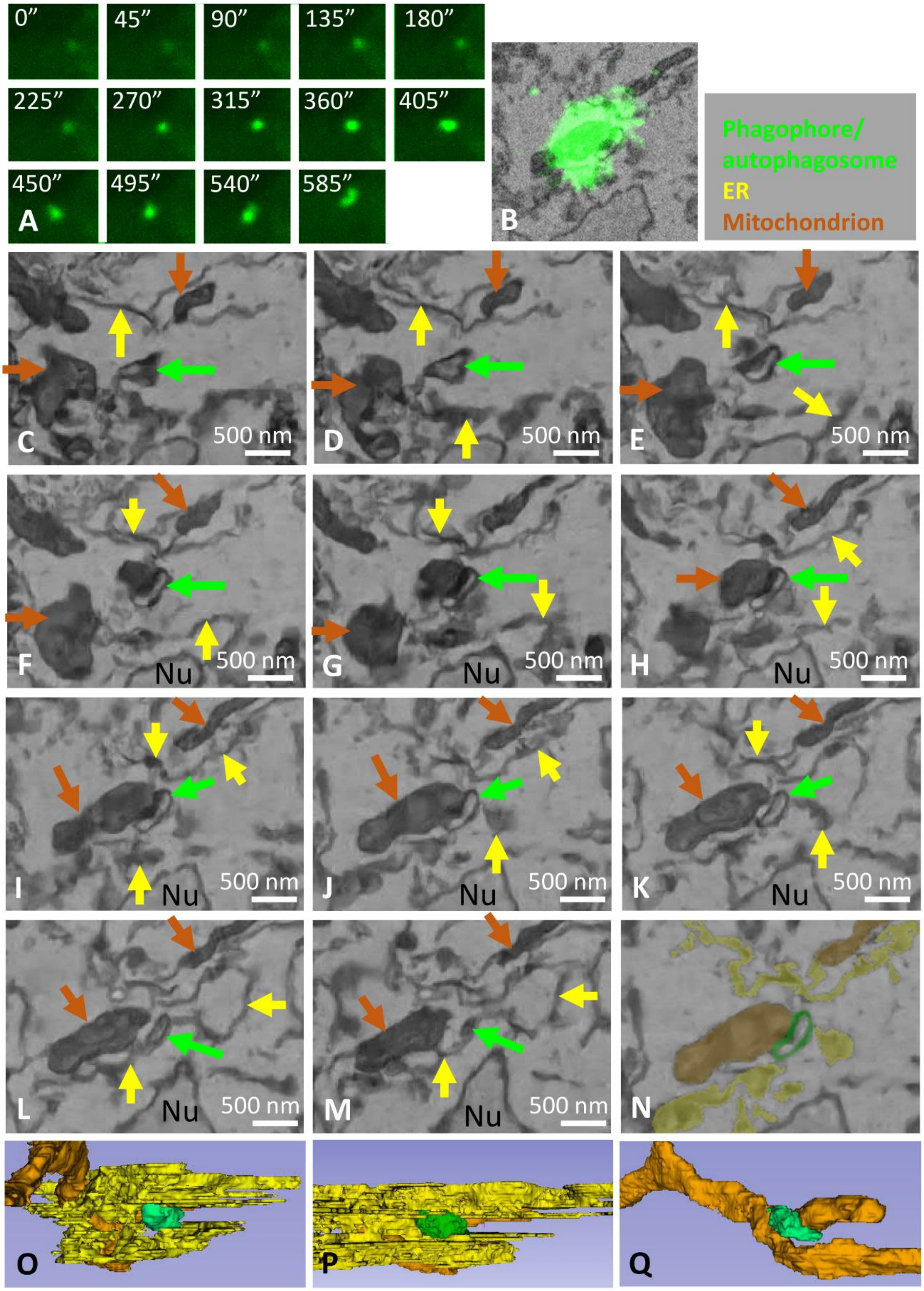

Supplementary Figure S2. CLEM of an omegasome and a nascent phagophore/autophagosome using SBEM. A. HEK293 cells expressing GFP-tagged DFCP1 were time-lapse imaged to trace omegasomes. The

cells were fixed 6 min 45 sec after the appearance of the omegasome. After fixation, the same cell and omegasome were imaged with SBEM. B. One image of the SBEM stack overlaid with the DFCP1 fluorescence. C-M. Images from the SBEM stack showing a phagophore/autophagosome (green arrows), ER (yellow arrows) and mitochondria (brown arrows). N. The phagophore, ER and mitochondria were traced to create a 3D model, using the color code shown at the upper right corner of the Figure. O-Q. Views of he 3D model from different angles, showing the relationships of the phagophore, ER and mitochondria with each other. Nu, nucleus. See also Supplementary Figure S1.

Suppl. Figure S3

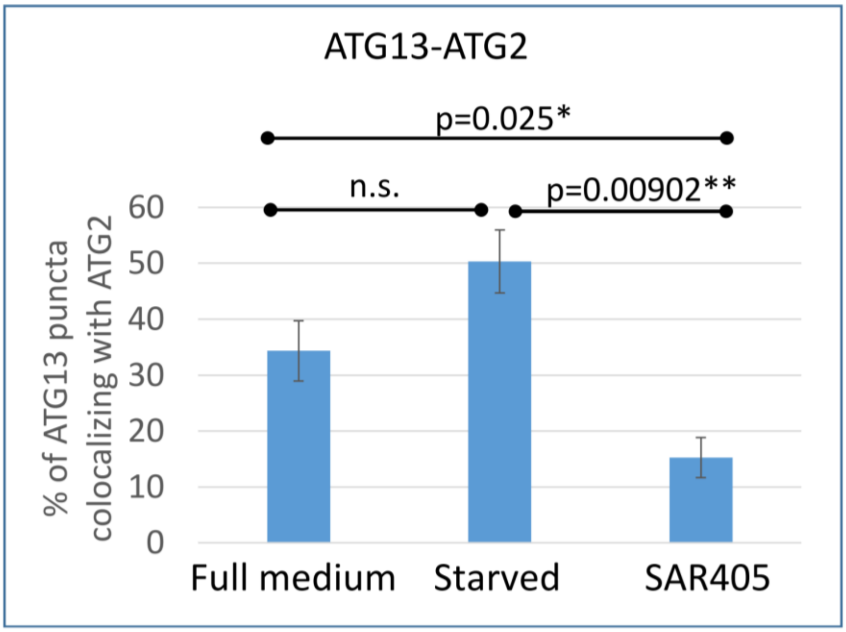

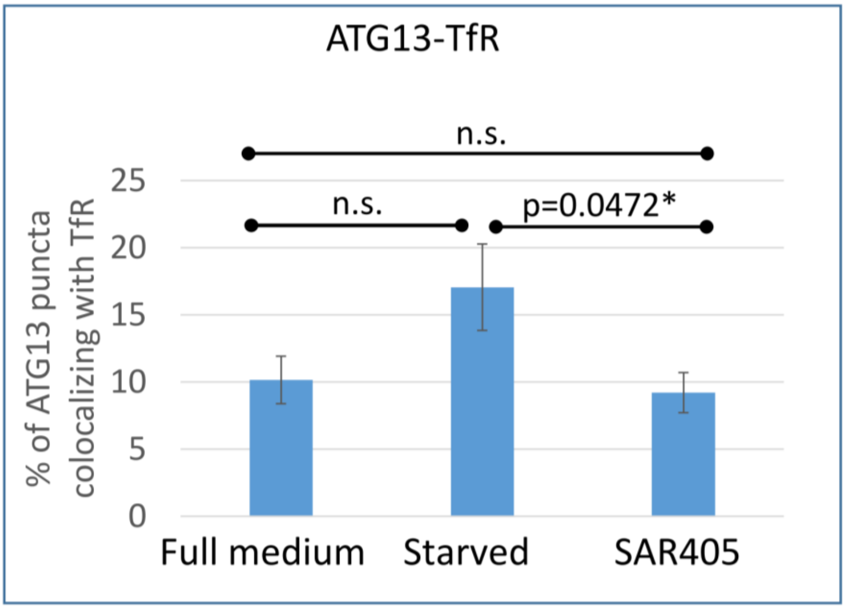

Supplementary Figure S3. Colocalization/proximal localization analysis of GFP-tagged ATG13 with endogenous ATG2 and TfR. HEK293 cells expressing GFP-tagged ATG13 were fixed without treatment, or after 60-min amino acid starvation without or with the PI3K inhibitor SAR405, which prevents autophagosome biogenesis. Endogenous ATG2 or TfR were labelled with immunofluorescence staining. GFP-ATG13 puncta with their epicenters located within 120 nm from the epicenters of puncta positive for ATG2 or TfR were quantified. The results are expressed as proportion of total ATG13 puncta locating in proximity of ATG2 or TfR positive puncta. Five independent experiments were performed (n=5). The error bars represent standard error of the mean. Statistical significance was analyzed using the non-parametric Kruskal-Wallis analysis of variance, followed by post-hoc Mann-Whitney test.

Suppl. Figure S4

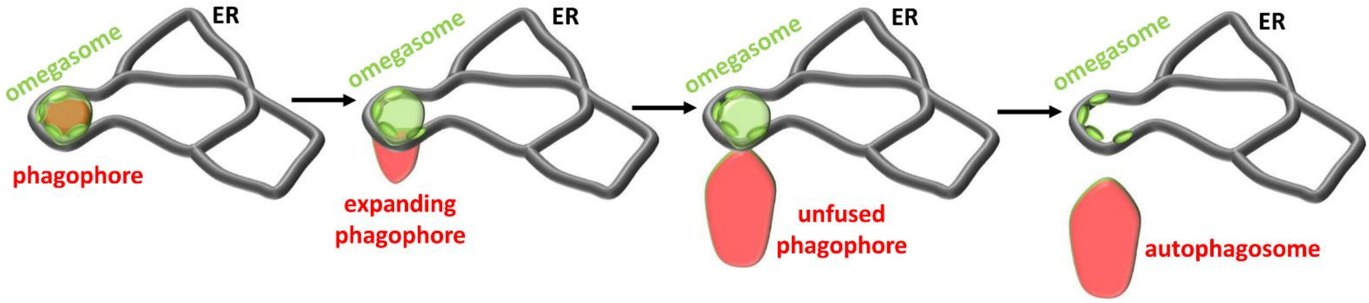

Supplementary Figure S4. Model of autophagosome biogenesis in the omegasome. The phagophore initiates, expands and closes into an autophagosome inside the omegasome. The omegasome collapses back to the ER once autophagome biogenesis has completed. The omegasome consist of specialized ER tubules and small sheets. See also Figure 1.
